# Protective Effect of *Chrysanthemum morifolium* cv. Fubaiju Hot-Water Extracts Against ARPE-19 Cell Oxidative Damage by Activating PI3K/Akt-Mediated Nrf2/HO-1 Signaling Pathway

**DOI:** 10.3389/fnut.2021.648973

**Published:** 2021-04-07

**Authors:** Yiming Hao, Yanfang Li, Jie Liu, Ziyuan Wang, Boyan Gao, Yaqiong Zhang, Jing Wang

**Affiliations:** ^1^China-Canada Joint Lab of Food Nutrition and Health (Beijing), Beijing Technology & Business University (BTBU), Beijing, China; ^2^Institute of Food and Nutraceutical Science, School of Agriculture and Biology, Shanghai Jiao Tong University, Shanghai, China

**Keywords:** *Chrysanthemum morifolium*, Fubaiju hot-water extracts, ARPE-19 cell, oxidative damage, PI3K/AKT, Nrf2/HO-1

## Abstract

*Chrysanthemum morifolium* cv. Fubaiju is a kind of widely consumed herb tea with multiple health benefits. The present study was aimed to evaluate the protective capacity of *C. morifolium* cv. Fubaiju hot-water extracts (CMs) against ARPE-19 cell oxidative damage. The results showed that pretreatment with 100 μg/mL CM could significantly reduce cell oxidative damage and apoptosis. Proapoptotic protein expression such as Bax, cleaved caspase-3, and cleaved poly(ADP-ribose) polymerase (PARP) was significantly decreased after CM addition, while the expression level of antioxidant enzymes including catalase, glutamate-cysteine ligase catalytic subunit (GCLc), superoxide dismutase 2 (SOD2), and NAD(P)H:quinone oxidoreductase 1 (NQO-1) was significantly promoted. Meanwhile, CM treatment upregulated Akt phosphorylation, nuclear factor erythroid 2-related factor 2 (Nrf2) nuclear translocation, and the expression level of antioxidant gene heme oxygenase-1 (HO-1) in a dose-dependent manner under oxidative stress. Knockdown of Nrf2 by targeted small interfering RNA (siRNA) alleviated CM-mediated HO-1 transcription and almost abolished CM-mediated protection against hydrogen peroxide (H_2_O_2_)-induced cell damage. Correspondingly, the protective effect of CM was dramatically blocked after interference with phosphatidylinositol 3-kinase (PI3K)/Akt inhibitor LY294002, indicating that the protective effect of CM on cell oxidative damage was attributed to PI3K/Akt-mediated Nrf2/HO-1 signaling pathway.

**Graphical Abstract:**
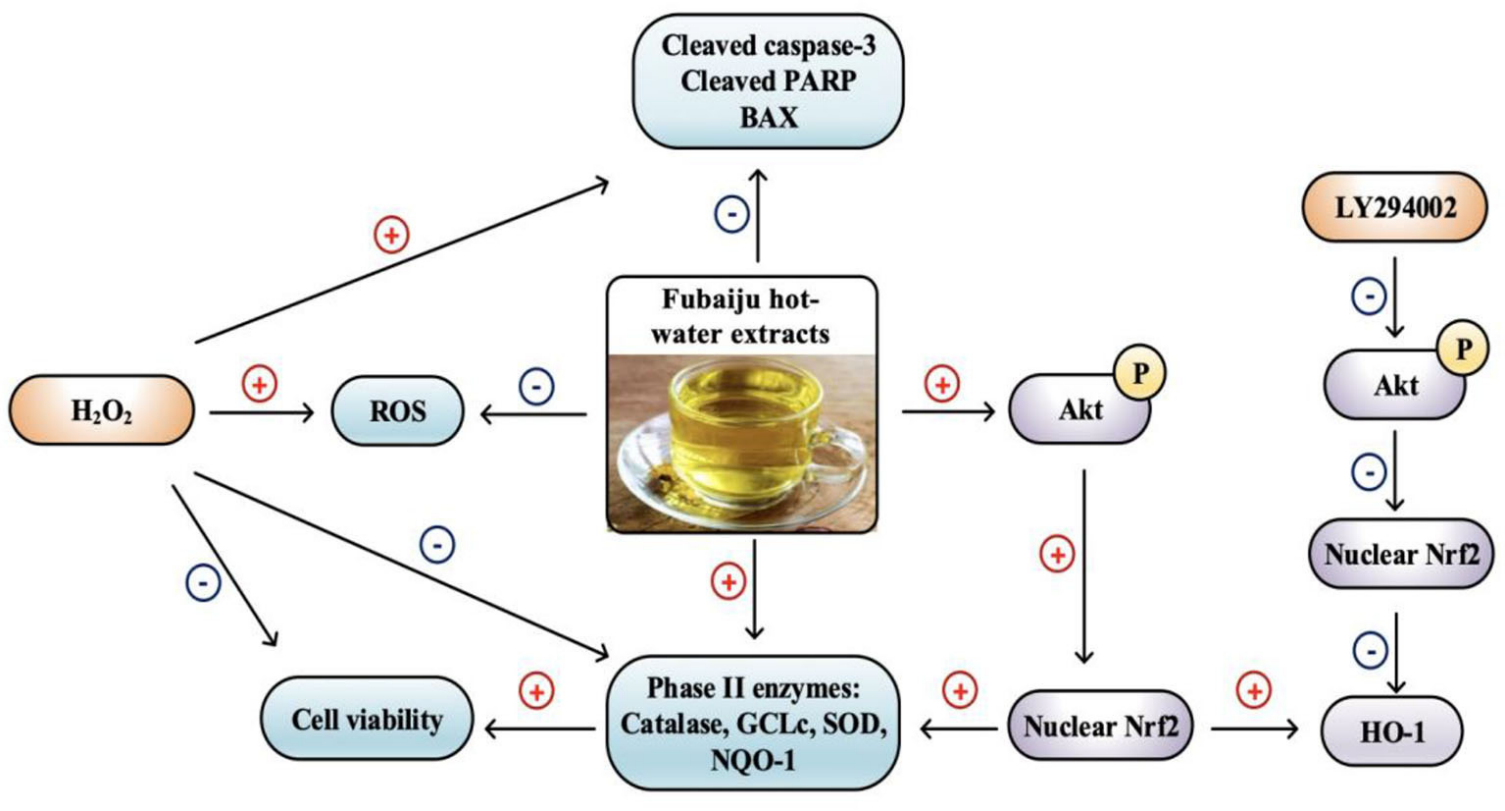
Fubaiju protected ARPE-19 cells oxidative damage by activating PI3K/Akt mediated Nrf2/HO-1 signaling pathway.

## Introduction

Age-associated macular degeneration (AMD) is the major cause of blindness among the elderly (1–3). Previous literature has indicated that early dietary intervention of natural antioxidants may be an effective and safe way to prevent AMD (4–6). For instance, it was reported that dietary enrichment with natural antioxidants from grape extracts prevented retinal pigment epithelium (RPE) oxidation, cytoskeletal damage, and vision loss in β5^−/−^ mouse (6). And it was found that grape extracts rich in polyphenols could restore the structure and visual function among pathologic visual decline patients (4, 5). AMD has been proven to be associated with oxidative damage, among which nuclear factor erythroid 2-related factor 2 (Nrf2) pathway has been involved to combat oxidative stress (7–10). After activation, Nrf2 translocates to the nucleus and binds with the antioxidant response element (ARE) to activate phase II antioxidant enzyme transcription, including catalase, glutamate-cysteine ligase catalytic subunit (GCLc), superoxide dismutase 2 (SOD2), NAD(P)H:quinone oxidoreductase 1 (NQO-1), and heme oxygenase-1 (HO-1). It has been found that wheat alkylresorcinols could protect ARPE-19 cells against hydrogen peroxide (H_2_O_2_)-induced oxidative damage *via* activation of Akt-dependent Nrf2/HO-1 signaling (10). And zeaxanthin was reported to increase Nrf2-mediated phase II enzyme expression through phosphatidylinositol 3-kinase (PI3K)/Akt activation for the prevention of ARPE-19 cell apoptosis induced by oxidative stress (11). On the contrary, Nrf2 deficiency could magnify the oxidative damage in RPE cells (12). Inhibition of Akt phosphorylation was reported to increase RPE cell oxidative damage and apoptosis (3, 10, 11). Thus, Akt-dependent Nrf2 signaling pathway activation might be a key target for the treatment of oxidative stress-induced retinal diseases.

*Chrysanthemum morifolium* Ramat. is a well-known food-medicine homologous flower tea that is rich in bioactive components such as flavonoids and phenolic acids (13–17). It has been reported that *C. morifolium* possesses many biological activities, including cardiovascular protection (18), antiallergy (19), gut microbiota modulation (20), anti-inflammation (14, 15), and antioxidant properties (14, 15). For instance, the hot-water extracts of *C. morifolium* could suppress lipopolysaccharide (LPS)-induced RAW 264.7 macrophage pro-inflammatory cytokine expression, such as interleukin-6 (IL-6), IL-1β, and cyclooxygenase-2 (COX-2) (14, 15). Our previous study also showed that different cultivars of chrysanthemums could protect ARPE-19 cell through depressing the increment of H_2_O_2_-induced intercellular reactive oxygen species (ROS) production (15). However, the mechanism of the protection of RPE cells from oxidative damage by chrysanthemum is still unclear at present. *C. morifolium* cv. Fubaiju was selected in the present study for its excellent bioactivity, great sensory quality, and large-scale production (15). The cytoprotective effects of *C. morifolium* cv. Fubaiju hot-water extracts (CMs) on H_2_O_2_-induced oxidative stress in ARPE-19 cells, along with the underlying mechanism, were investigated. Results of this study could provide a theoretical basis for the application of Fubaiju as bioactive ingredients in dietary supplements for the potential treatment of AMD and promote the local economy.

## Materials and Methods

### Chemicals and Reagents

*C. morifolium* cv. Fubaiju samples were obtained from Hubei Mingmu Health Technology Co. Ltd., China. The 2′,7′-dichlorofluorescin diacetate (DCFH-DA) and H_2_O_2_ were obtained from Sigma-Aldrich (St. Louis, MO, USA). LY294002 and the antibodies against α-tubulin, Nrf2, HO-1, Akt, p-Akt, catalase, GCLc, NQO-1, cleaved caspase-3, cleaved poly(ADP-ribose) polymerase (PARP), Bax, Bcl-2, and Histone H3 were purchased from Abcam (Cambridge, UK). SOD2 was ordered from CST (MA, USA). Control and Nrf2 small interfering RNA (siRNA) (sc-37007 and sc-37030) were ordered from Santa Cruz Biotechnology (Dallas, TX). Milli-Q purification system (Millipore Laboratory, Bedford, MA) was applied for ultrapure water preparation.

### Preparation of Fubaiju Hot-Water Extracts

CMs were prepared following a reported procedure (15). Ground Fubaiju sample (2 g) was added into boiling water (20 mL) and left at room temperature overnight prior to centrifugation. Then, supernatant (5 mL) was collected and dried by freeze-dryer, and the residue was separated and weighed, followed by redissolving in H_2_O (5 mL) and 10 times dilution with H_2_O. The diluent was injected to ultra-performance liquid chromatography combined with a quadrupole time-of-flight mass spectrometer (UPLC/Q-TOF-MS) to investigate its chemical composition. The chemical composition of CM was composed of 26 compounds including 21 flavonoids and five phenolic acids, in which apigenin-7-O-glucoside, kaempferol-3-O-acetyl-glucoside, and apigenin-7-O-acetylglucoside were the three major components (14).

### Cell Culture

Human retinal pigment epithelial cell line (ARPE-19 cells) was obtained from ATCC and cultured as previously reported (21). Briefly, ARPE-19 cells were cultured in Dulbecco's modified Eagle's medium (DMEM)/F12 medium supplemented with 10% fetal bovine serum, 100 U/mL penicillin, and 100 μg/mL streptomycin (Gibco, Madison, USA) at 37°C containing 5% CO_2_. Before hydrogen peroxide treatment, cells were cultured in serum-free DMEM/F12 medium for 30 min.

### Cell Viability Assay

The ARPE-19 cell viability was evaluated with the 3-(4,5-dimethylthiazol-2-yl)-2,5-diphenyltetrazolium bromide (MTT) assay as previously reported (22). ARPE-19 cells (1 × 10^4^ cells/well) were seeded in 96-well plates and cultured for 24 h, followed by incubation with CM for 12 h, then treated with H_2_O_2_ for another 24 h. After MTT treatment (0.5 mg/mL, Sigma Aldrich, St. Louis, MO, USA) for another 4 h, crystal violet was dissolved in dimethyl sulfoxide (DMSO) and the absorbance at 490 nm was measured by Tecan microplate spectrophotometer (Spark 10M, Tecan, Männedorf, Switzerland).

### Intracellular Reactive Oxygen Species Measurement

Intracellular ROS was measured according to the laboratory protocol (15). Briefly, the ARPE-19 cells (1 × 10^4^ cells/well) were seeded in 96-well black plates for 24 h followed by pretreatment with CM for another 24 h. Then, cells were rinsed with Hanks' balanced salt solution (HBSS) and treated with DCFH-DA (10 μM) for 30 min at 37°C, followed by 24-h incubation with H_2_O_2_ (300 μM) in serum-free medium. Fluorescence intensity was examined using microplate reader with excitation at 475 nm and emission at 530 nm.

### Western Blotting

Western blotting was performed as reported before (23). Briefly, to prepare the whole cell protein, ARPE-19 cells from different treatment groups were harvested and extracted with ice-cold radioimmunoprecipitation assay (RIPA) buffer mixed with phosphatase inhibitors and protease inhibitor cocktail. To extract nuclear and cytoplasmic protein, relevant nuclear and cytoplasmic protein extraction kits (Beyotime, Shanghai, China) were applied. Bicinchoninic acid (BCA) protein assay kit (Thermo Fisher, Madison, USA) was used for protein concentration determination. To separate the target proteins, protein samples (40 μg) were subjected to 12% polyacrylamide gel electrophoresis and electro-transferred to polyvinylidene fluoride (PVDF) membranes (Bio-Rad Laboratories, Hercules, USA) at 1 A, 25 V conditions for 30 min. Skim milk (5% v/v) in Tris-buffered saline containing 0.05% Tween-20 (TBST) was used for blocking at room temperature for 1.5 h. Then, target proteins were detected by incubation with corresponding primary antibodies overnight at 4°C following incubation (2 h) with horseradish peroxidase-linked secondary antibodies at ambient temperature. The protein bands were visualized using chemiluminescence reagent (Bio-Rad Laboratories, Hercules, USA). In addition, α-tubulin and Histone H3 were selected as loading control for total protein and nuclear protein, respectively.

### Nrf2 SiRNA Knockdown

ARPE-19 cells (1 × 10^5^ cells/well) were plated in 12-well plates for 24 h, following transfection with 100 nM of control siRNA or Nrf2 siRNA for 12 h (Lipofectamine 2000, Thermo Fisher, Madison, USA). Subsequently, the cells were incubated with 100 μg/mL CM for another 12 h prior to treatment with 400 μM H_2_O_2_. The cytoprotective effects of different treatments were evaluated by MTT assay.

### Statistical Analysis

Data were indicated as means ± SD (*n* = 3). The comparisons between different groups were conducted by one-way ANOVA and analyzed by Duncan's test using SPSS 19.0 software. The value of *P* ≤ 0.05 was statistical significance.

## Results

### *Chrysanthemum morifolium* cv. Fubaiju Hot-Water Extracts Prevented H_2_O_2_-Induced ARPE-19 Cell Oxidative Damage and Intracellular Reactive Oxygen Species Production

**Figure 1:**
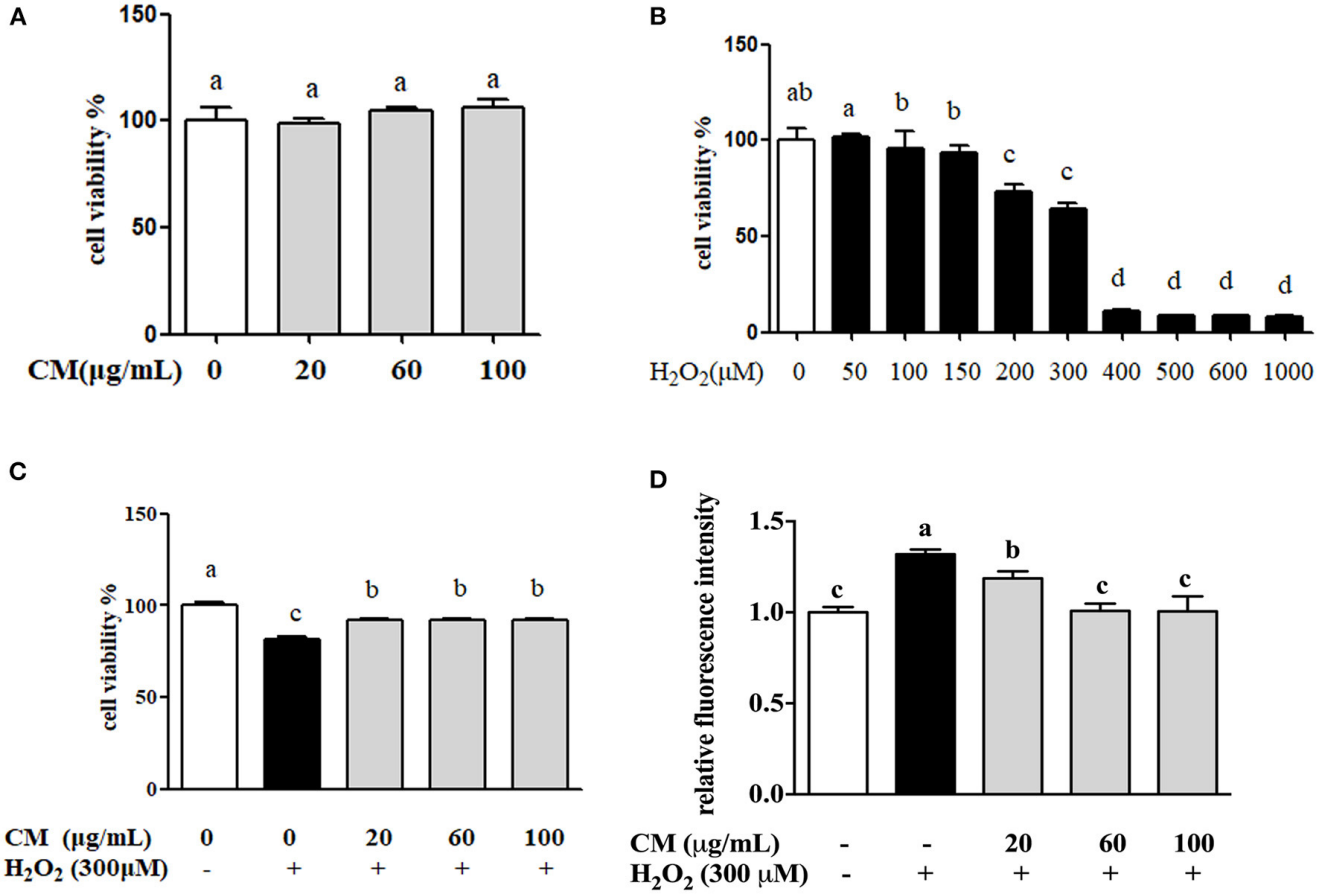
The protective effects of Fubaiju hot-water extracts (CMs) on hydrogen peroxide (H_2_O_2_)-induced ARPE-19 cell oxidative damage and reactive oxygen species (ROS) generation. **(A)** The cytotoxicity of CM on ARPE-19 cells. **(B)** The cell viability of ARPE-19 cells induced by different doses of H_2_O_2_. **(C)** CM treatment inhibited H_2_O_2_-induced ARPE-19 cell damage. **(D)** The suppression of CM on ROS production in ARPE-19 cells. The results are mean ± SD (*n* = 3). Columns with different letters are significantly different (*P* < 0.05).

MTT results showed that there was no significant difference of cell viability between the cells treated with different doses of CM (20, 60, and 100 μg/mL) and the control group (Figure 1A). The cell viability of ARPE-19 cells was significantly decreased in a dose-dependent manner after incubation with H_2_O_2_, and 300 μM H_2_O_2_ was chosen to use in subsequent experiments (Figure 1B). As shown in Figure 1C, pretreatment with different concentrations of CM could attenuate 300 μM H_2_O_2_-induced decrease of cell viability. Iloki-Assanga et al. (24) reported that excessive intracellular ROS generation could induce severe oxidative damage and lead to chronic degenerative and ocular diseases. Meanwhile, different concentrations of CM (20, 60, and 100 μg/mL) showed a dose-dependent inhibitive effect on intracellular ROS production (Figure 1D). Here, 300 μM H_2_O_2_ treatment significantly increased the intracellular ROS production by 31.9% compared to that of the control group, while ROS generation was significantly decreased by 10.0, 23.5, and 23.8% after 20, 60, and 100 μg/mL CM treatment, respectively (Figure 1D; *P* < 0.05). These results suggested that the CM-mediated cytoprotective activity might be related to the inhibition of ROS generation.

### *Chrysanthemum morifolium* cv. Fubaiju Hot-Water Extracts Decreased Proapoptotic Related Protein Expression

**Figure 2:**
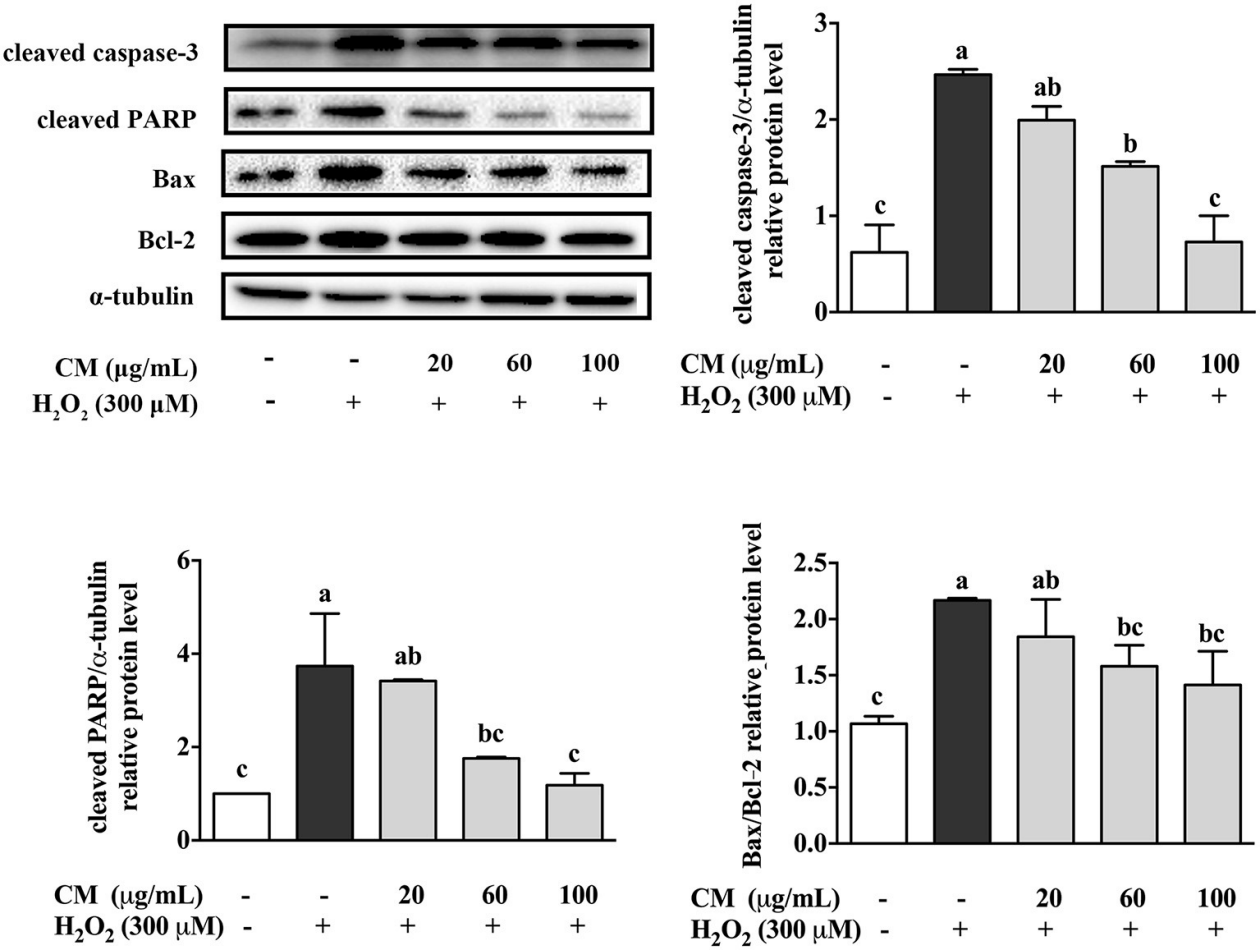
Effects of Fubaiju hot-water extracts (CMs) on apoptosis-related protein expression induced by hydrogen peroxide (H_2_O_2_) in ARPE-19 cells. After pretreatment with 20, 60, and 100 μg/mL CM for 24 h, the cells were then co-treated with 300 μM H_2_O_2_ for another 4 h. The protein expression levels of cleaved caspase-3, cleaved poly(ADP-ribose) polymerase (PARP), Bax, Bcl-2, and α-tubulin were determined by Western blotting. Here, α-tubulin was used as a loading control. The protein levels of the bands were quantified by densitometry. The results are mean ± SD (*n* = 3). Columns with different letters are significantly different (*P* < 0.05).

The apoptosis-related protein expression of cleaved caspase-3, cleaved PARP, Bax, and Bcl-2 was evaluated through Western blotting. Cleaved caspase-3 and cleaved PARP are major biomarkers in apoptosis (25, 26). Bax was evidenced to increase the release of cytochrome *c* from mitochondria to trigger apoptosis, while Bcl-2 reversed these effects *via* stabilizing the mitochondrial membrane to block cytochrome *c* release (27). Compared with the control group, treatment with 300 μM H_2_O_2_ notably increased the expression level of cleaved caspase-3 and cleaved PARP and the ratio of Bax/Bcl-2 (Figure 2; *P* < 0.05). In contrast, after pretreatment with CM, the increased expression of these proapoptotic related proteins was markedly suppressed in a dose-dependent manner (Figure 2; *P* < 0.05). The ratio of Bax/Bcl-2 was an important predictive indicator of cell apoptosis, which was positively associated with apoptosis (27). Pretreatment with CM significantly decreased the H_2_O_2_-induced upregulation of cleaved caspase-3, cleaved PARP, and Bax/Bcl-2 ratio, indicating that CM could prevent ARPE-19 cells from H_2_O_2_-induced apoptosis. Considering that cell apoptosis could be induced by the accumulation of ROS under oxidative stress (28), the antiapoptotic effect of CM could be closely related to its antioxidant capacity against oxidative stress.

### *Chrysanthemum morifolium* cv. Fubaiju Hot-Water Extracts Increased Antioxidant Enzyme Protein Expression

**Figure 3:**
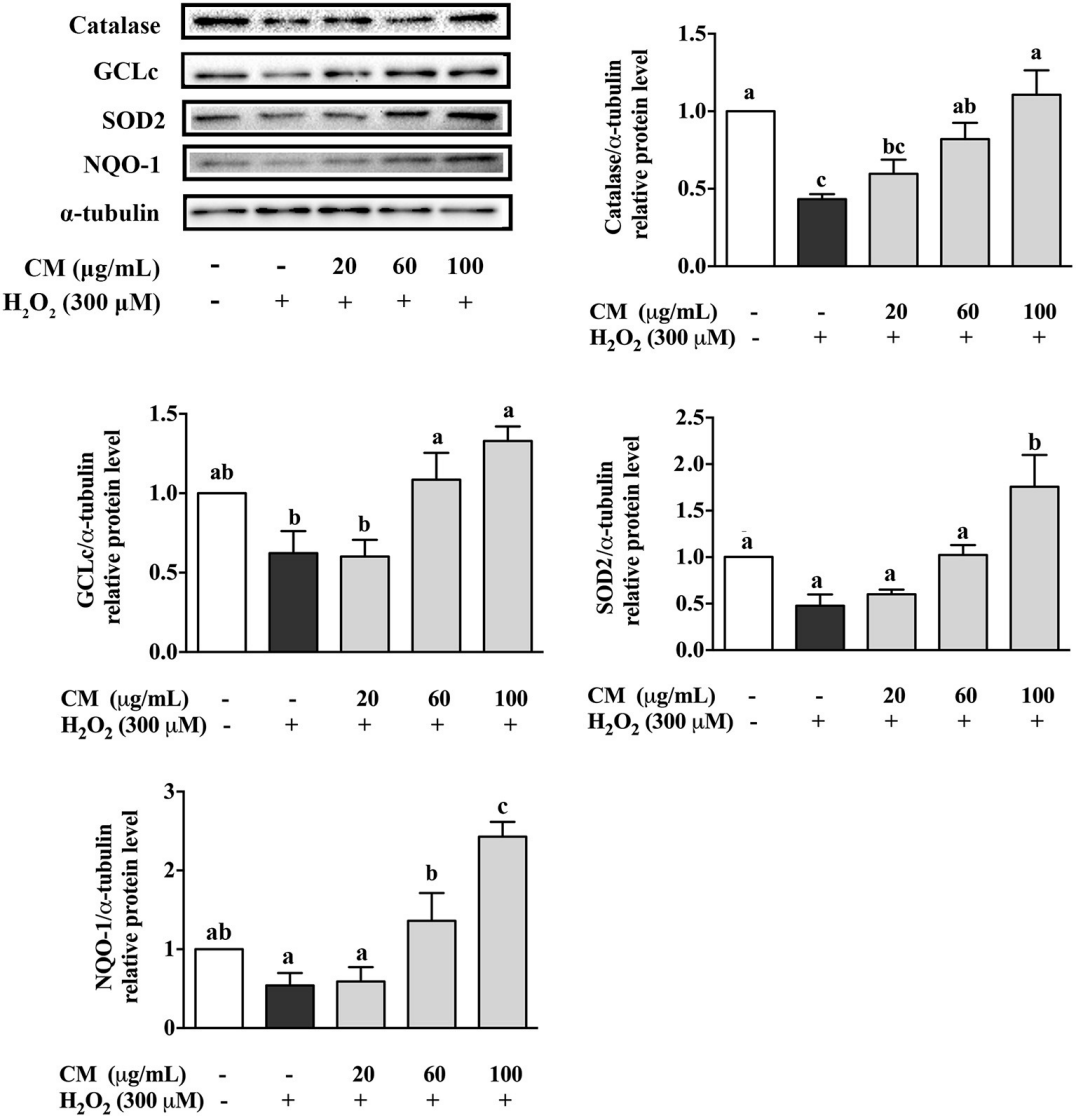
Effects of Fubaiju hot-water extracts (CMs) on antioxidant enzyme protein expression induced by hydrogen peroxide (H_2_O_2_) in ARPE-19 cells. After pretreatment with 20, 60, and 100 μg/mL CM for 24 h, the ARPE-19 cells were then co-treated with 300 μM H_2_O_2_ for another 4 h. The protein levels of catalase, glutamate-cysteine ligase catalytic subunit (GCLc), superoxide dismutase (SOD), and NAD(P)H:quinone oxidoreductase 1 (NQO-1) were determined by Western blotting using the corresponding antibodies. Here, α-tubulin was used as a loading control. The protein levels of the bands were quantified by densitometry. The results are mean ± SD (*n* = 3). Columns with different letters are significantly different (*P* < 0.05).

To investigate the antioxidative mechanisms of CM against H_2_O_2_-induced cell damage, the protein expression levels of antioxidant enzymes including catalase, GCLc, SOD2, and NQO-1 were detected by Western blot. H_2_O_2_ treatment notably decreased the expression of catalase, GCLc, SOD2, and NQO-1 (Figure 3; *P* < 0.05). However, compared to the H_2_O_2_ group, pretreatment with 100 μg/mL CM remarkably enhanced the expression of catalase, GCLc, SOD2, and NQO-1 by 155.2, 113.4, 267.6, and 347.8%, respectively (Figure 3; *P* < 0.05). The results indicated that catalase, GCLc, SOD2, and NQO-1 might be involved in the protective role of CM against H_2_O_2_-induced oxidative stress.

### *Chrysanthemum morifolium* cv. Fubaiju Hot-Water Extracts Attenuated H_2_O_2_-Induced Oxidative Damage Through the Nrf2/HO-1 Signaling Pathway

**Figure 4:**
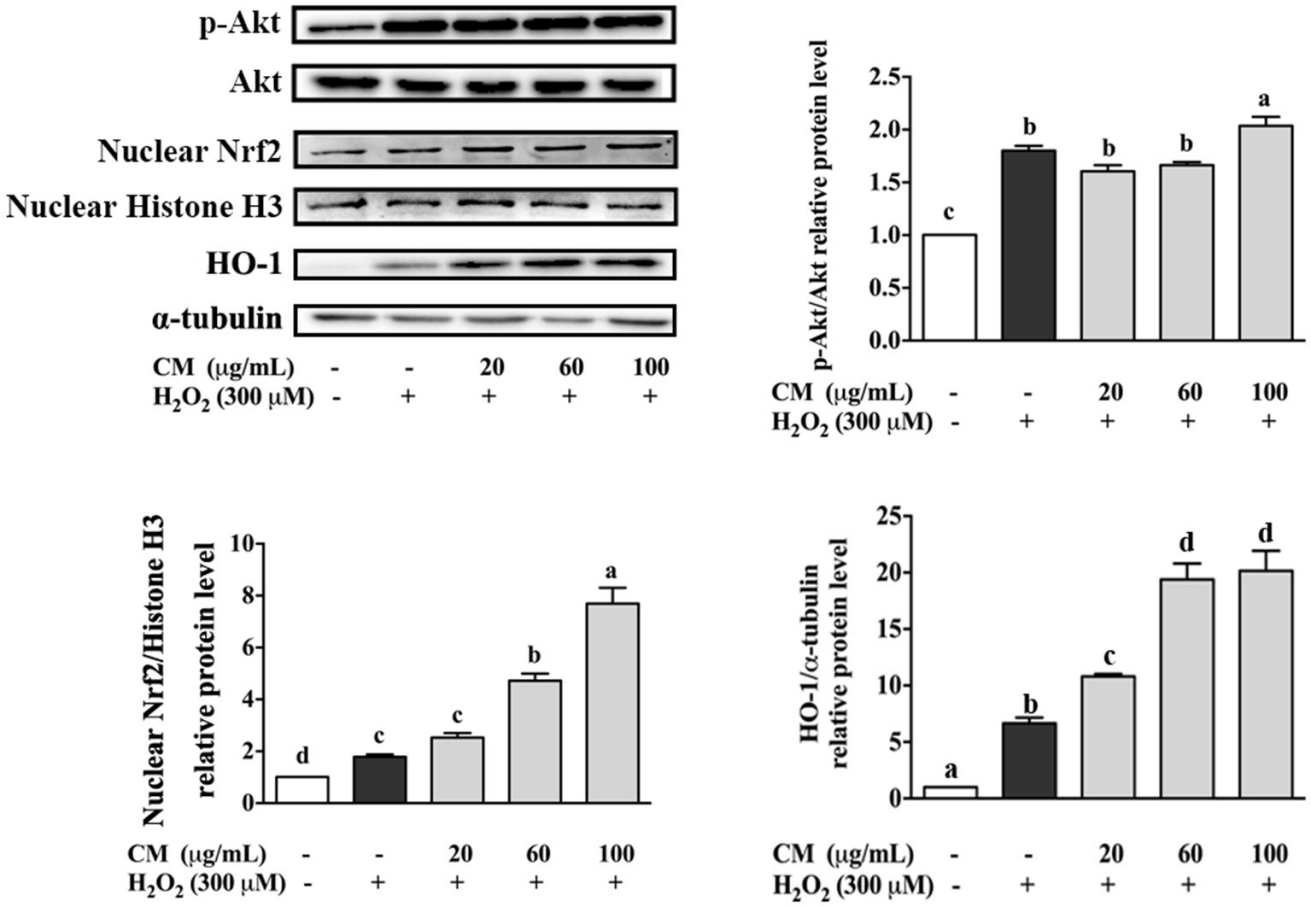
Effects of Fubaiju hot-water extracts (CMs) on Akt/nuclear factor erythroid 2-related factor 2 (Nrf2) signaling pathway-related protein expression induced by H_2_O_2_ on ARPE-19 cells. After pretreatment with 20, 60, and 100 μg/mL CM for 24 h, the cells were then treated with 300 μM H_2_O_2_ for another 0.5 h. The protein levels of phosphor (*p*) or total (*t*) Akt, nuclear Nrf2, nuclear Histone H3, heme oxygenase-1 (HO-1), and α-tubulin were determined by Western blotting. The results are mean ± SD (*n* = 3). Columns with different letters are significantly different (*P* < 0.05).

**Figure 5:**
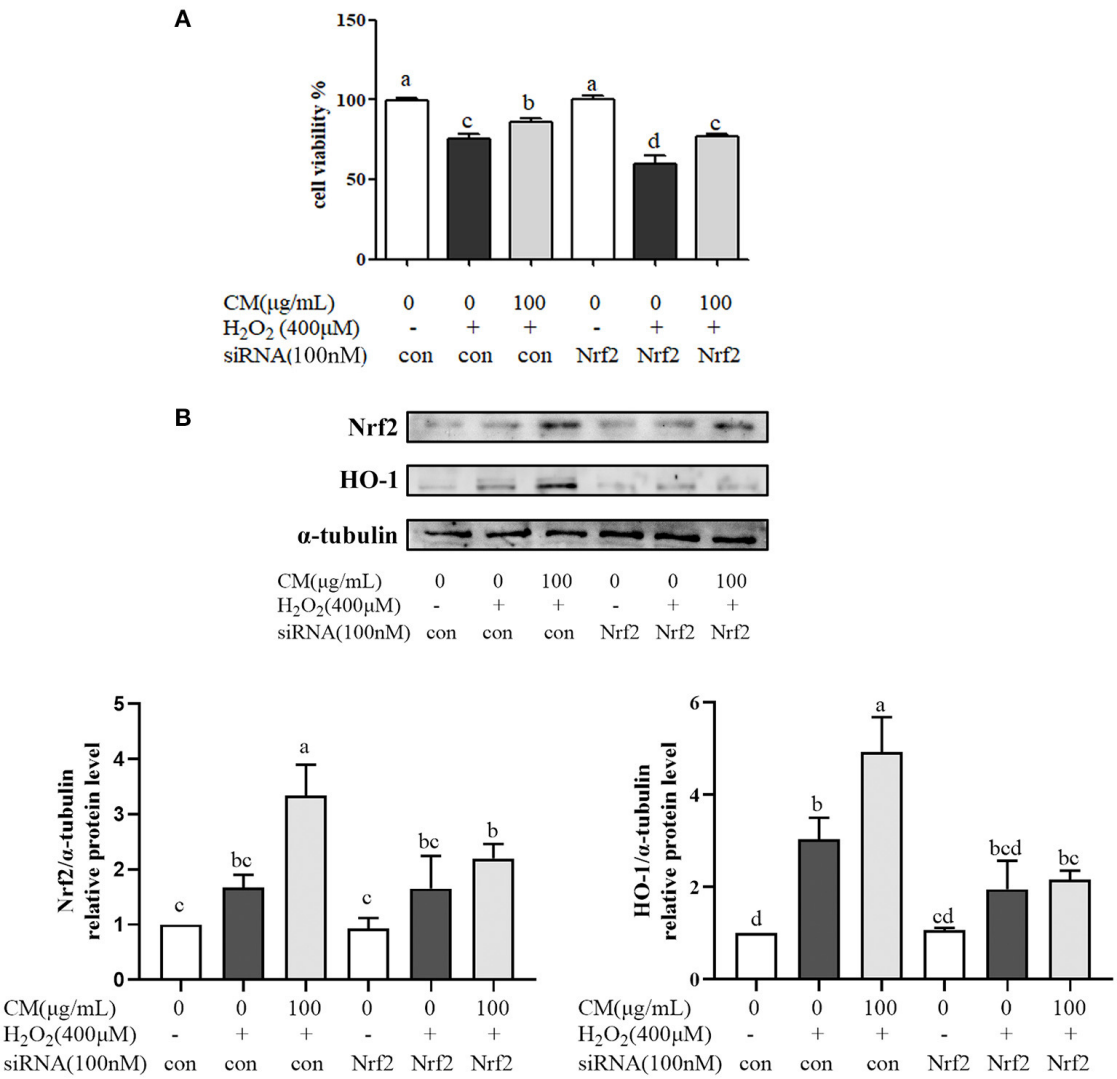
Fubaiju hot-water extracts (CMs) protected against hydrogen peroxide (H_2_O_2_)-induced ARPE-19 cell oxidative damage *via* activating the nuclear factor erythroid 2-related factor 2 (Nrf2)/heme oxygenase-1 (HO-1) pathway. ARPE-19 cells were transfected with or without 100 nM Nrf2 small interfering RNA (siRNA) for 12 h and then treated with or without 100 μg/mL CM for another 12 h before incubation with 400 μM H_2_O_2._ **(A)** The cytoprotective effect of CM on ARPE-19 cells induced by H_2_O_2_ with control or Nrf2 siRNA. **(B)** The protein levels of Nrf2, HO-1, and α-tubulin were determined by Western blotting. Here, α-tubulin was used as a loading control. The protein levels of the bands were quantified by densitometry. The results are mean ± SD (*n* = 3). Columns with different letters are significantly different (*P* < 0.05).

The effects of CM on the expression of Akt and nuclear Nrf2 and HO-1 were detected by Western blot. In our present study, the phosphorylation of Akt was markedly increased by pretreatment with 100 μg/mL CM compared with the group treated with 300 μM H_2_O_2_ (Figure 4; *P* < 0.05). PI3K/Akt signaling pathway is important for the activation and nuclear translocation of Nrf2 (7, 8, 29). The transcription factor Nrf2 regulates the expression of important cytoprotective enzymes, such as HO-1 (28). CM treatment elevated the expression of nuclear translocated Nrf2 and its downstream HO-1 in a dose-dependent manner (Figure 4). To verify whether Nrf2 was required for the protective function of CM, Nrf2 was silenced using siRNA interference (Figure 5A). Cell viability decreased significantly after incubation with 400 μM H_2_O_2_ compared to the untreated group (Figure 5A; *P* < 0.05), while 100 μg/mL CM remarkably reversed the decrement, which was consistent with the abovementioned results (Figure 1C). Treatment with 100 nM Nrf2 siRNA showed no effect on cell viability in comparison to the untreated group. However, pretreatment with 100 nM Nrf2 siRNA could aggravate H_2_O_2_-induced cell damage and weaken the cytoprotective effects of CM compared to the counterpart groups without Nrf2 siRNA treatment (Figure 5A). In addition, preincubation with 100 nM Nrf2 siRNA significantly abolished CM-mediated upregulation of Nrf2 and HO-1 at the protein level (Figure 5B; *P* < 0.05), suggesting that Nrf2 was required for the protection of CM on ARPE-19 cell oxidative damage.

### *Chrysanthemum morifolium* cv. Fubaiju Hot-Water Extracts Suppressed ARPE-19 Cell Damage Through the PI3K/Akt-Mediated Nrf2/HO-1 Signaling Pathway

**Figure 6:**
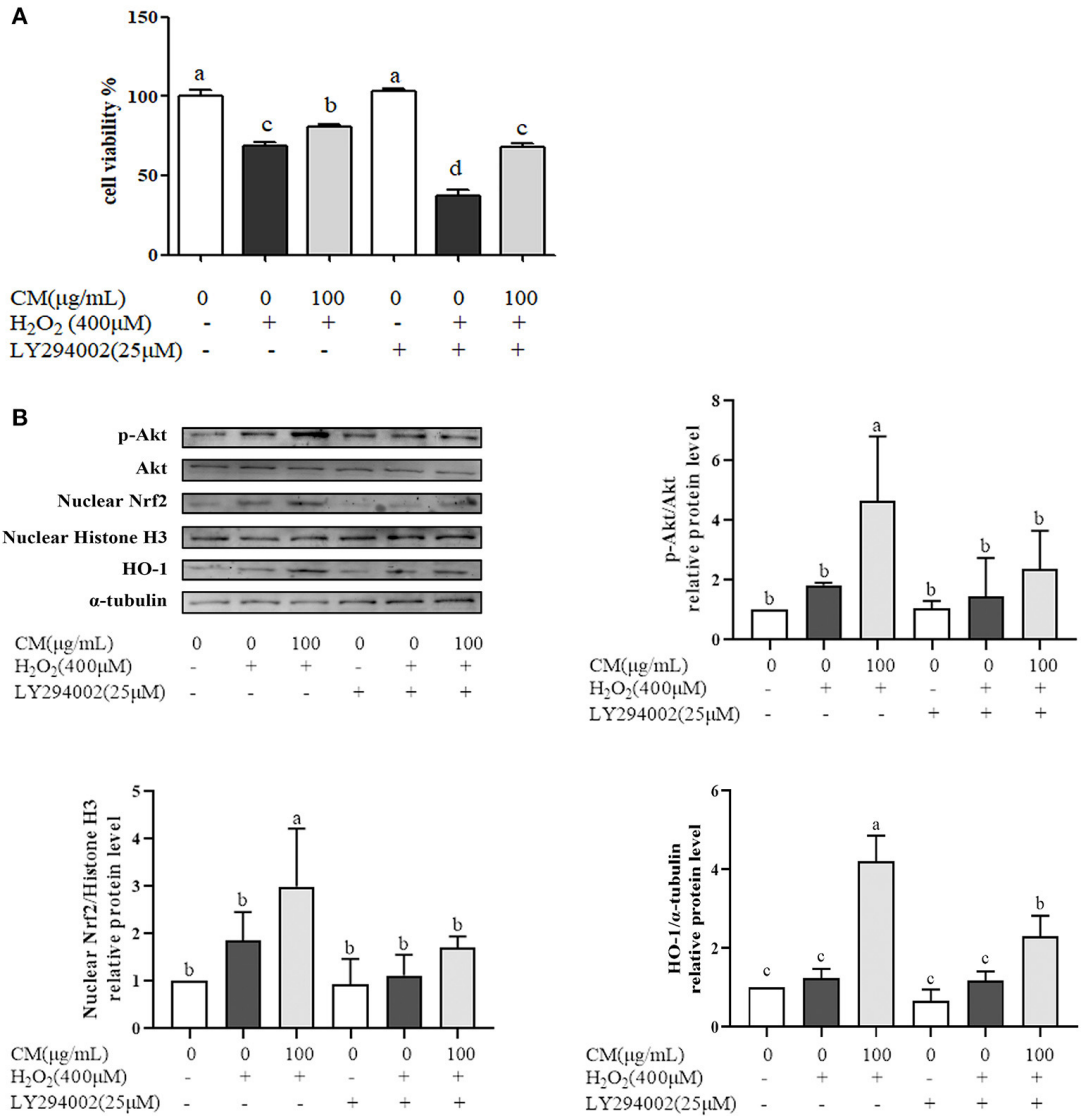
Fubaiju hot-water extracts (CMs) protected against hydrogen peroxide (H_2_O_2_)-induced ARPE-19 cell oxidative damage by activating the Akt-dependent nuclear factor erythroid 2-related factor 2 (Nrf2)/heme oxygenase-1 (HO-1) pathway. ARPE-19 cells were cultured with 100 μg/mL CM for 24 h and then transfected with or without 25 μM phosphatidylinositol 3-kinase (PI3K) inhibitor LY294002 for 4 h before incubation with or without 400 μM H_2_O_2_. **(A)** The cytoprotective effect of CM on ARPE-19 cells induced by H_2_O_2_ with or without LY294002. **(B)** The protein levels of phosphor (*p*) or total (*t*) Akt, nuclear Nrf2, nuclear Histone H3, HO-1, and α-tubulin were determined by Western blotting. The protein levels of the bands were quantified by densitometry. The results are mean ± SD (*n* = 3). Columns with different letters are significantly different (*P* < 0.05).

Nrf2/HO-1 signaling pathway could be activated *via* activation of PI3K/Akt pathway (30). PI3K inhibitor (LY294002) was used to identify whether PI3K/Akt pathway was activated for the protection of CM on ARPE-19 cells (Figure 6). Treatment with LY294002 alone had no effect on the cell viability compared with the non-treatment group; however, pretreatment with LY294002 could aggravate cell damage caused by H_2_O_2_ and impair the protective effects of CM compared to the counterpart group without LY294002 treatment (Figure 6A; *P* < 0.05). Moreover, LY294002 remarkably blocked the CM-mediated phosphorylation of Akt and decreased its downstream gene expression, such as nuclear translocated Nrf2 and HO-1 at the protein level (Figure 6B), implying the involvement of PI3K/Akt pathway in the protection of CM against H_2_O_2_-induced ARPE-19 cell oxidative damage.

## Discussion

This study revealed the underlying mechanism of the cytoprotection of CM on H_2_O_2_-induced oxidative stress in ARPE-19 cells. Oxidative stress induces RPE cell apoptosis, which is the major contributor to the onset and development of many age-related degenerative diseases (29, 31). Our results suggested that CM treatment could reduce intracellular ROS production and promote phase II enzyme expression such as catalase, GCLc, SOD2, NQO-1, and HO-1, causing the reduction of ARPE-19 cell apoptosis. Previous study also indicated CMs were able to scavenge DPPH, ABTS^+^, and oxygen radicals; depress the increment of H_2_O_2_-induced intercellular ROS production; and inhibit the expression of LPS-induced inflammatory cytokines (14, 15). The possible reason for the protective effects of CM against oxidative stress in ARPE-19 cells may be because it is rich in natural antioxidants. A total of 26 compounds including 21 flavonoids and five phenolic acids were identified from CM, in which apigenin-7-O-glucoside, kaempferol-3-O-acetyl-glucoside, and apigenin-7-O-acetylglucoside were the three major components (14–16).

To further reveal the underlying mechanism of the cytoprotective activity of CM, Nrf2 activation and the expression of downstream antioxidant enzymes were investigated. Nrf2 is a redox-sensitive transcription factor that regulates many phase II antioxidant enzyme expression, and activation of Nrf2 is evidenced to be one of the critical defensive mechanisms against oxidative stress (32). Our results demonstrated that transfecting ARPE-19 cells with Nrf2 siRNA accelerated H_2_O_2_-induced oxidative damage and depressed the protective effects of CM against oxidative stress. In line with the above results, apigenin could attenuate the tert-butyl hydroperoxide (t-BHP)-induced oxidative damage in ARPE-19 cells *via* the Nrf2 pathway (33). The activities of SOD, catalase, glutathione peroxidase (GSH-PX), and the total antioxidant capacity (T-AOC) were notably increased after apigenin intervention (33). Natural bioactive compounds in food ingredients could promote Nrf2 activation and protect the retina from oxidative stress causing retinal diseases. For example, it was found that kaempferol prevented ARPE-19 cells from H_2_O_2_-induced oxidative damage and apoptosis *via* reducing ROS production and decreasing the expression of Bax/Bcl-2, caspase-3, and vascular endothelial growth factor (VEGF) (34). Weng et al. (35) reported that quercetin restored the H_2_O_2_-reduced cell viability decrement and increased Nrf2, NQO-1, and HO-1 expression at the protein level. The phytochemicals of CM included many multi-bioactive compounds such as aglycone and glycoside of kaempferol, quercetin, and apigenin, which might partly explain that the possible mechanism of CM against oxidative stress in ARPE-19 cells was through Nrf2 signaling activation and its downstream antioxidant enzyme expression. However, whether any synergistic interaction occurs between these compounds is still unclear, which requires more research in the future. Moreover, previous research has demonstrated that PI3K/Akt pathway plays a critical role in modulating Nrf2/HO-1 protein expression as an upstream signaling, which is crucial for the cytoprotection of RPE cells (7). PI3K/Akt could make Nrf2 dissociate from Keap1 and facilitate subsequent signal transduction to induce the activation of antioxidant enzymes (36). There are many studies demonstrating that continuous oxidative stress will lead to cell damage *via* the downregulation of PI3K/Akt signaling pathway (37). Thus, upregulation of PI3K/Akt might play an important role against RPE cell oxidative damage. For example, piceatannol could protect ARPE-19 cells against H_2_O_2_-induced oxidative stress and apoptosis through modulating PI3K/Akt/Nrf2/HO-1 signaling pathway (7). Our present study indicated that PI3K inhibitor (LY294002) counteracted CM-mediated increase of Akt phosphorylation, Nrf2 nuclear translocation, HO-1 expression, and cytoprotective activity, which was in accordance with other published studies (3, 10, 11), suggesting that CM activated Akt-dependent Nrf2 signaling to protect ARPE-19 cells from oxidative stress.

## Conclusions

In conclusion, we found that CMs were non-toxic for ARPE-19 cells and could significantly reduce H_2_O_2_-induced oxidative damage and apoptosis. The cytoprotective effects of CM against oxidative stress were *via* activating the PI3K/Akt-mediated Nrf2/HO-1 pathway. These results could offer an extended window for the utilization and consumption of Fubaiju as food-medicine homologous functional flower tea in functional foods/beverages to minimize the risk of AMD. However, the identification and characterization of the specific bioactive substance in Fubaiju that plays the vital role still need further study.

## Data Availability Statement

The original contributions generated for this study are included in the article/supplementary material, further inquiries can be directed to the corresponding author/s.

## Author Contributions

YH contributed to writing the original draft preparation, data curation and analysis, and software and investigation. YL contributed to writing the original draft preparation, data curation, and analysis. JL contributed to writing, reviewing, and editing, supervision, and project administration. ZW contributed to writing, reviewing, and editing. BG and YZ contributed to the conceptualization and methodology. JW contributed to the supervision and project administration. All authors contributed to the article and approved the submitted version.

## Conflict of Interest

The authors declare that the research was conducted in the absence of any commercial or financial relationships that could be construed as a potential conflict of interest.
